# Integrating piezoresistive sensors on the embouchure analysis of the lower lip in single reed instrumentalists: implementation of the lip pressure appliance (LPA)

**DOI:** 10.1002/cre2.214

**Published:** 2019-09-05

**Authors:** Miguel Pais Clemente, Joaquim Mendes, Joana Cerqueira, André Moreira, Mário Vasconcelos, Afonso Pinhão Ferreira, José Manuel Amarante

**Affiliations:** ^1^ INEGI, Labiomep Faculty of Medicine Porto Porto Portugal; ^2^ INEGI, Labiomep, Faculty of Engineering University of Porto Porto Portugal; ^3^ Faculty of Dental Medicine Porto Porto Portugal; ^4^ Department of Dental Biomaterials, Faculty of Dental Medicine University of Porto Porto Portugal; ^5^ Department of Orthodontics, Faculty of Dental Medicine University of Porto Porto Portugal

**Keywords:** clarinet, embouchure, ethylene‐vinyl acetate, forces, lip pressure appliance, mouthpiece, piezoresistive sensors, saxophone

## Abstract

**Background:**

It is essential to understand, characterize, and measure the embouchure mechanism of a wind instrumentalists, where the applied forces on the perioral tissues can usually promote discomfort or pain.

**Methods:**

The sample consisted of five clarinet players and five saxophone players. The embouchure force measurements at the lower lip area were assessed using a piezoresistive sensor (FlexiForce^TM^, Tekscan, Boston, USA, 0.07 kgf/cm^2^) placed on the lower part of the mouthpiece of the single reed instrument. Furthermore, each participant performed three times three different notes at different pitches: high, medium, and low. An intraoral device was manufactured in order to dissipate the existing pressures.

**Results:**

The piezoresistive sensors applied to the mouthpiece of the five clarinetists presented values between 16 and 226 g of force. In the case of the five saxophonists, the values registered were between 5 and 320 g of force.

**Conclusions:**

Piezoresistive sensors are a valid option to characterize that single reed instrumentalists apply substantial forces at the lower lip that can be equivalent to medium orthodontic forces. The implementation of the Lip Pressure Appliance can be a valid solution on the prevention of eventual lesions resulting from the embouchure forces.

**List of Abbreviations**
LPA: lip pressure appliance
EVA: ethylene‐vinyl acetate

## 1 BACKGROUND

Wind instrument players and string instrument players carry out their musical activity within high physical and cognitive demands (Kotani & Furuya, 2018; Nedelcut, Leucuta, & Dumitrascu, 2018; Viljamaa, Liira, Kaakkola, & Savolainen, 2017). Their body, more precisely their musculoskeletal system, has to accomplish the exigent, challenging, and rigorous positions adopted during their musical performance (Fasshauer, Frese & Evers, 2015; Liu & Hayden, 2002). The brain of these musicians acts as a computer, where the mathematical rhythm of the music score results in the production of a melodic sound recognized as music. Nevertheless, within this procedure that seems simple, it is important to understand, characterize, and measure the muscular motion, the forces applied, and the mandibular kinematics of each musician throughout their musical activity. In performing arts medicine, the usefulness of sensor devices are imperative because we can analyze and quantify the inherent activity of the musician that is directly linked to the orofacial biomechanics of his performance. From a medical point of view, this can provide clinicians to gather valid information, in order to be able to fulfill a correct diagnoses and enhance a better treatment plan when handling with this specific population.

Single reed instrumentalists like saxophone or clarinet player promote a retrusion of the lower lip over the lower anterior incisors during their embouchure and musical performance. The embouchure mechanism is super‐sensitive to any disturbing influence, therefore it is necessary to promote a great comfort in order to have an efficient performance. The player when disturbed may have his ‘tone’ affected and limit his scope for artistic interpretation (Porter, 1973). It is interesting to notice that some of these instrument players have adopted a solution of placing a cigarette rolling folded paper over the lower central incisors in order to avoid the appearance of any discomfort or pain on the mucosa of the lower lip. Besides traumatic or pressure ulcers, other type of lesions may be present in the oral cavity. Oral vesiculoerosive diseases that have an immunopathic cause, such as lichen planus, pemphigus vulgaris, mucous membrane pemphigoid, erythema multiform, and recurrent aphthous stomatitis, also manifest oral ulcers. Moreover, oral herpetic lesions that can be primary or recurrent can arouse a typical small group of ulcers (Hargitai, 2018). In single reed instrumentalists, the occurrence of traumatic lesions or ulcers on this region is a possibility that can occur due to the high pressures that are induced by the mouthpiece of the single reed instrument players. Nevertheless, it is natural to observe that not every saxophonist or clarinetist uses the paper wrapper to protect the single reed instrumentalist from undesired pressures. This fact is normally associated to the musical teachers and their teaching techniques, because many clarinet and saxophone professional advocate that the students need to feel the pressures of their instrument during musical performance. The circumstance of using the cigarette rolling folded paper over the mandibular incisors could eventually allow the musical students to apply higher and unnecessary forces during their embouchure. Likewise, one of the most common citations among musicians is “no pain, no gain.” In this particular research, the authors intend to characterize and understand the phenomena of the embouchure of single reed instrument players, within a specific issue that is for these musicians a major concern, the forces applied to the lower lip, and the discomfort or pain that can be felt within this region. However, it is important to understand that there are specific features and characteristics of the dental occlusion, teeth rotations, and crowding teeth of the single reed instrument player that can be detrimental for the occurrence of more or less discomfort on the lower lip after playing clarinet or saxophone.

The aim of this study is the measurement of the medium and maximum applied forces at the lower lip during the embouchure mechanism of single reed instrumentalists and the implementation of an intraoral device that may dissipate the existing pressures.

## 2 METHODS

### 2.1 Study population

The subjects were selected from the Superior School of Music and Performing Arts of Porto, Portugal, where they were taking there master of science degree in music performance, on the class of clarinet and saxophone. The sample consisted of five clarinet players (group C) and five saxophone players (group S). They were included in this study with the following criteria: they were absent of pain in the orofacial structures, namely, the lower lip, and had no previous orthodontic treatment.

### 2.2 Implementation of piezoresistive sensors to characterize embouchure forces

**Figure 1:**
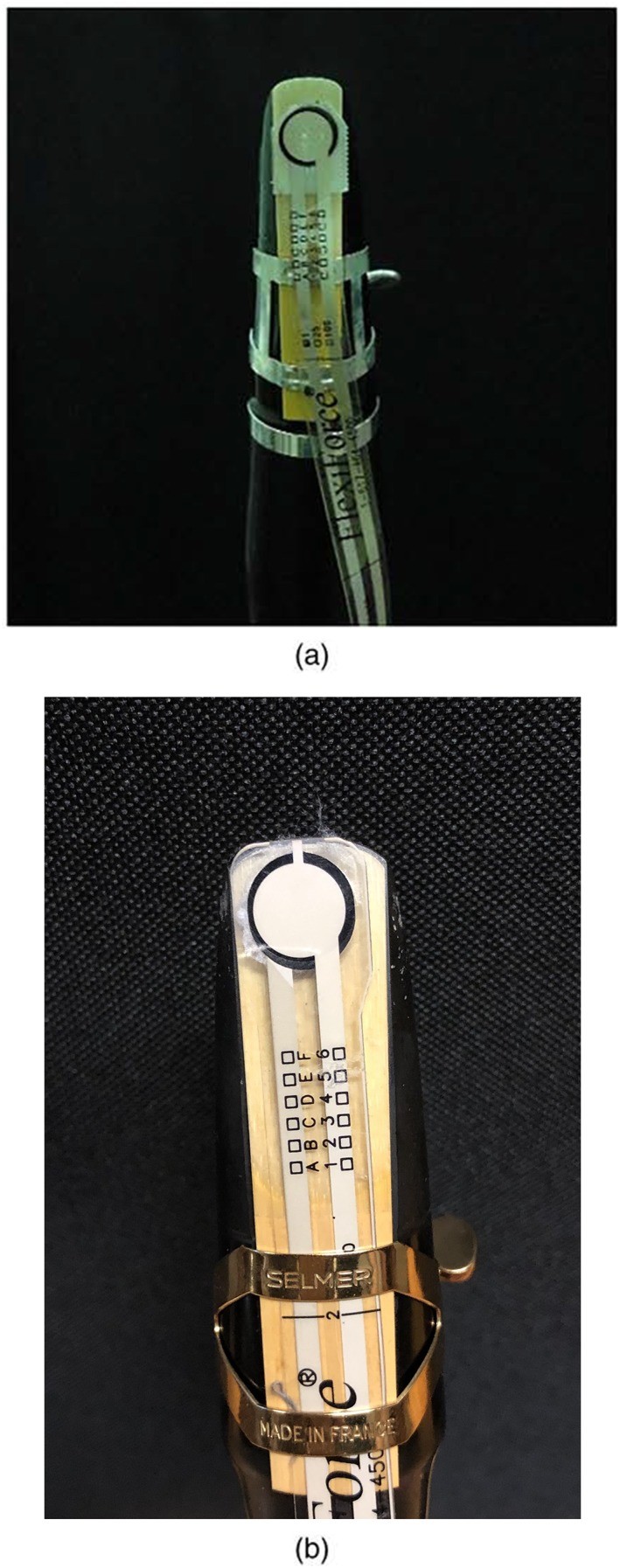
Piezoresistive sensor placed under the mouthpiece of the clarinet (a) and saxophone (b) [Correction added on 30 October 2019, after Online publication: Figure 1‐b has been updated in this current version.]

In order to understand the forces that were applied in these wind instrumentalists during their embouchure, on the lower anterior incisal block, the piezoresistive sensors were applied on the lower part of the mouthpiece. The embouchure force measurements at the lower lip area were assessed using one piezoresistive sensor (FlexiForce^TM^, Tekscan, Boston, USA,70 gf/cm^2^). The sensor was previously calibrated and integrated with the manufacturer's recommendations. For calibration, four different weights (100, 250, and 500 and 1000 g) were placed on the piezoresistive sensor, and the voltage output was observed with a data acquisition board and LabVIEW 2011 (National Instruments, Austin, USA). The piezoresistive sensor was placed in the lower part of the mouthpiece of the single reed instruments in order to assess the embouchure mechanism of each participant (Figure 1). Furthermore, each participant performed three times three different notes at different pitches: high, medium, and low. The average of medium and maximum pressure was obtained from the nine essays.

### 2.3 Manufacture of the Lip Pressure Appliance (LPA)

**Figure 2:**
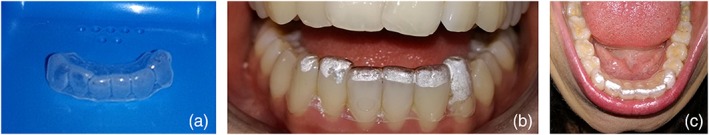
LPA (a); LPA placed on the six anterior mandibular teeth, from canine to canine, frontal view (b) and occlusal view (c)

The LPA is made from a thermoformable sheet of EVA (ethylene‐vinyl acetate) with only 1‐mm thickness. For the manufacture of the LPA, a mandibular impression was done with alginate to obtain the respective dental cast. The LPA design is limited to the área of the six mandibular anterior teeth (Figure 2), involving the canines, the lateral incisors and the central incisors. The contour of this customized mouthpiece is predetermined to the clinical crowns of the respective teeth til the interdental papilla both on the vestibular and lingual surface. Before starting the thermoforming of the LPA, the working cast is hydrated for 5 min to facilitate the removal of the LPA. The Erkoform 3D Motion (Erkodent®, Pfalzgrafenweiler, Deutschland) was used for an automatic and standard thermoforming with respect to the thickness and geometry of the plate, time of heating, and cooling. For this particular plate, a 160°C heating was needed before the plate was placed over the dental cast automatically. The thermoforming process was done regarding the plate properties cooling phase during the adaption of the thermoforming material to the dental cast. This step can last for 45 seconds and at this point the thermoforming sheet will be removed from the dental cast and trimmed by the area previously chosen. Before delivering the LPA to the instrumentalist, a correct polishing and finishing is done with a felt disc (Scotch‐Brite^TM^) at a 10.000 to 15.000 rpm. The main objective of this step is to smooth the surfaces and to relieve papilla areas. In single reed instrumentalists the use of tobacco shrouds is usual. This is a cheap and easy solution for single reed instrumentalists to protect their lower lip. The embouchure at this class of wind instruments is done by stabilizing the instrument between the upper central incisors and the retruded lower lip that covers the mandibular incisors. Thus, consequently, the lower lip will induce a pressure against the incisal border of the central and lateral incisors. After long periods of practice this can often result in the presence of discomfort, pain and even traumatic ulcers at the lower lip, therefore the LPA was implemented in this population of single reed instrumentalists.

## 3 RESULTS

Table 1 shows the results obtained from the piezoresistive sensors applied to the mouthpiece of the five clarinetists that were part of the present study. The median values registered were between 16 and 120 g, and the maximum values registered were between 25 and 226 g of force.

**Table 1:** Maximum and medium values of force registered in the interface of the mouthpiece and the lower lip of the clarinetists, Group C

	C.F.	D.S.	J.V.	L.M	R.L.
Maximum values of force (g)	25	59	51	129	226
Medium values of force (g)	16	42	33	83	120

Table 2 shows the results obtained from the piezoresistive sensors applied to the mouthpiece of the five saxophonists that were part of the present study. The median values registered were between 5 and 169 g, and the maximum values registered were between 22 and 320 g of force.

**Table 2:** Maximum and medium values of force registered in the interface of the mouthpiece and the lower lip of the saxophonists, Group S

	C.F.	D.R.	G.S.	J.O.	J.P.G.
Maximum values of force (g)	80	22	320	220	60
Medium values of force (g)	42	5	169	127	27

The placement of the LPA in the mouth seemed to be imperceptible. The feedback given by the musicians after using the LPA was satisfactory since they refer that this device doesn't interfere with the sound quality produced.

## 4 DISCUSSION

The whole complex of anatomical structures around the mouth and the way they are used for playing the wind instrument is called the “embouchure.” The three major components of embouchure are the tongue, the teeth, and the muscles of cheek and lip (van der Weijden, Kuitert, Berkhout, & van der Weijden, 2018). Although the saxophone and the clarinet belong to the same family of wind instruments, the single reed, they have different mouthpieces that will require a specific technique to form the embouchure, each individual has a specific orofacial structure and masticatory function; therefore, individuals will develop their own embouchure mechanism (Yeo, Pham, Baker, & Porters, 2002). Theoretically, both upper central incisors should stabilize the mouthpiece equally during the performance of saxophonist and clarinetist together with the keratinized (exterior) part of the lower lip. However, some participants, showed that there is usually a “predominant” tooth where greater pressure will be applied during the embouchure mechanism (Clemente et al., 2018). A recent systematic review of van der Weijden et al (2018) concluded that tooth position may influence musical performance and embouchure comfort of wind instrumentalists and that an extreme malocclusion could interfere with the wind instrumentalists' performance (van der Weijden et al., 2018).

We may verify from the present results that most of the participants perform their embouchure applying a medium force greater than 50 g on the lower lip. Additionally, during musical practice, fluctuations in the pressure can occur during their performance. Thus, maximum values may be significantly higher than the median values, reaching pressures such as 300 g. If we take into consideration that the average of the median values of the five clarinet players is 58.8 g of force on the lower lip and the saxophonists can reach 94 g of force, it should be highlighted the importance of a customized intraoral device that can help to dissipate the high pressures that are being exerted by the mouthpiece on the lower lip against the teeth.

Wind instrument players are aware of the physical problems that can affect certain certain parts of their body like arms, shoulders, or cervical region. For these musicians it is esential to be capable of dealing with a high demanding profession in this particular case being a saxophonist or a clarinetist were it is normal to have a stressful life, with emotions that can lead to certain states of anxiety. Therefore the knowledge of wind instrumentalists regarding the physical and psychological issues is a reality; nevertheless, to some extent usually, the orofacial region is sometimes underestimated, and few or no special care is taken by these musicians. As an example, if we think that a clarinet player always has a callus on his finger caused by the pressure and weight of the instrument when holding the instrument while performing. In fact it is possible to make a comparison with the perioral structures where the occurrence of a legion on the lower lip can easily occur due to the high pressures induced when playing a wind instrument like the saxophone or the clarinet. A musician that has tooth rotation, crowding of the lower incisors, or even a restoration done on one of the teeth where the lower lip is in contact can be a reason for the presence of orofacial pain or discomfort. Understanding the importance that these issues have in the musician's embouchure is fundamental in order to provide valid solutions for a better musical performance (Caetano, Bernardes, Twillert, Clemente, & Gabriel, 2017). In fact, it is true that any type of intraoral device that is placed can be awkward and unpleasant, so this research describes the manufacturing process of the LPA. The majority of the pressures applied to the orofacial structures while playing single reed instruments can be considered as a medium force. Taking into consideration that professional musicians play numerous hours per day reaching up to 6–8 hr of practice, this could be considered as an intermittent orthodontic force.

A study involving a population of rats has shown that tooth movement occurs with intermittent forces, although in a less magnitude than that in a continuous force (Kumasako‐Haga, Konoo, Yamaguchi, & Hayashi, 2009). A systematic review conducted by Ren et al (2003) gathered over than 400 studies on humans and animal experiments concerning the optimal force or range of forces for orthodontic tooth movement. A wide range of initial forces (18–1,500 cN) were described in literature. They found three studies suggesting an optimal continuous force from 50 to 200 cN for tipping premolar and 100 to 500 cN for tipping molars (Ren, Maltha, & Kuijpers‐Jagtman, 2003). Along with the possibility of occurring tooth movement, wind musicians may usually suffer other consequences of their profession, such as temporomandibular disorders (TMD), myospasms, hear loss, musculoskeletal disorders, and trauma associated to the upper and lower lips muscles (Nishiyama & Tsuchida, 2016; Sayegh Ghoussoub et al., 2008; Sousa, Machado, Greten, & Coimbra, 2017). Mucosal pressure ulcers and erosions are frequent in single reed wind instrumentalists, like saxophonists and clarinetists. This common lesion normally appears at the lower lip as result of the embouchure mechanism, where the vestibular surface of the teeth and the incisal edges are covered by the lower lip when performing the embouchure to produce a sound. When present, mucosal pressure ulcers on the lip can deteriorate the comfort of playing and in some instances turn impossible to execute some specific exercises (Phillips, 1972). To stabilize the mouthpiece, saxophonists and clarinetist are taught to use the lower lip and the upper central incisors; however, the constant and repeating contact between the incisal edges of the upper anterior teeth with the lip mucosa can consequently create erosions and ulcerations accompanied by severe pain (Bluj‐Komarnitka, Komarnitki, & Olczak‐Kowalczyk, 2014). It is comprehensive that when involved in a musical career, being a saxophonist and/or a clarinetist, stopping or diminishing the number of rehearsal hours is not a choice. Thus, musicians came up with a simple solution to keep playing and to decrease the incidence of ulcerations and erosions of the lower lip with the placement of tobacco shrouds folded over the lower incisors. This solution apparently works in satisfactory manner; nevertheless, some wind instrument players refer that at the end of the concert, they can be "spitting paper in their mouths". This can occur since the saliva will dissolve the sheet of paper, so the development of this lip pressure appliance seems to be a promising solution (Clemente et al.,^2007^). Moreover in the past some attempts have been made in order to highlight the importance of using and intra‐oral device to reduce the forces transmitted to the anterior central incisors (Porter, 1967). These previous work has the merit of introducing a subject that is a major concern for many wind instrumentalists, like the possibility of occurring tooth mobility, altered tooth positions with influence on the occlusion within the central incisors associated to the embouchure mechanism. For this purpose the authors referred the possibility of using a chrome cobalt casting framework when playing the instrument in order to promote the stabilization of the teeth that are exposed to such forces (Fine, 1986). During the clinical examination of some single reed instrumentalists that were included on our research, some referred that using an intra‐oral device such as the LPA with 1 mm of thickness could be "strange", so imagine a solution of removable partial denture, however this previous solution was mentioned in the 1980's. Nowadays, with the technological advancement it is possible to use thermoforming units, like the Erkoform 3d‐motion, were a reduction in the final of the material thickness can occur after the thermoforming process providing more comfort to the musician when using the customized LPA. In this pilot, study it was possible to quantify the forces that are transmitted by the mouthpiece of the saxofone and the clarinet on the lower lip which is retruded over the lower incisors, therefore the implementation of LPA is recommended.

## 5 CONCLUSIONS

Piezoresistive sensors were able to evaluate the forces applied by the single reed instrumentalists at the lower lip which can be equivalent to medium orthodontic forces. The development of the LPA can be a preventive approach to reduce the forces between the mouthpiece and the lower lip during musical performance. This intraoral device is removable, easy to manufacture and with a fabrication process that doesn't involve high costs. Dental clinicians should be aware of this option when observing a wind instrumentalist, especially in the case of saxophone players because these promote greater pressures on the lower lip when compared with the clarinet players. Likewise, musical teachers being aware of these issues can have an important role on the prevention of orofacial lesions and counseling student population.

## ETHICS APPROVAL AND CONSENT TO PARTICIPATE

Investigation approved on 9th January 2017 by the Ethics Committee of the Dental Medicine Faculty of the University of Porto, no. 000019.

## CONSENT FOR PUBLICATION

Not applicable.

## AVAILABILITY OF DATA AND MATERIALS

The datasets used and/or analyzed during the current study are available from the corresponding author on reasonable request.

## COMPETING INTERESTS

The authors declare that they have no competing interests.

## AUTHOR'S CONTRIBUTIONS

Conceptualization, M.P.C. and A.P.F.; Methodology, A.P.F.; Software, M.V.; Validation, J.G., J.M.A and A.P.F.; Formal Analysis, A.M.; Investigation, J.C.; Resources, J.G.; Data Curation, A.M.; Writing‐Original Draft Preparation, M.P.C.; Writing‐Review & Editing, A.M., M.P.C.; Visualization, J.G.; Supervision, A.P.F., J.G.; Project Administration, M.P.C and J.M.A.
